# Efficacy of UV-C 254 nm Light and a Sporicidal Surface Disinfectant in Inactivating Spores from *Clostridioides difficile* Ribotypes In Vitro

**DOI:** 10.3390/pathogens13110965

**Published:** 2024-11-05

**Authors:** Khald Blau, Claudia Gallert

**Affiliations:** Department of Microbiology–Biotechnology, Faculty of Technology, University of Applied Sciences Emden/Leer, 26723 Emden, Germany; khald.blau@hs-emden-leer.de

**Keywords:** *Clostridioides difficile*, UV-C light, sporicidal, ribotypes, disinfectant, sporicidal surface wipes, spores

## Abstract

*Clostridioides difficile* is widely recognised as one of the most common causes of healthcare-associated *C. difficile* infections due to the ability of spores to survive for prolonged periods in the hospital environment. This study aimed to evaluate the efficacy of UV-C 254 nm light in the inactivation of the spores of different *C. difficile* ribotypes on brain heart infusion (BHI) agar plates or in phosphate-buffered saline (PBS) with varying spore densities. Furthermore, the effectiveness of a sporicidal surface disinfectant against *C. difficile* spores was determined on different surfaces. Spore suspensions of different *C. difficile* strains in the range of 10^5^–10^7^ colony-forming units (CFUs) mL^−1^ were inoculated on BHI agar plates or in PBS and exposed to UV-C light for up to 30 min. Additionally, a spore suspension of 10^3^–10^5^ CFUs was spread over a 1 cm^2^ test area on different surfaces, and sporicidal surface wipes were used according to the manufacturer’s instructions. The findings demonstrated that spores of *C. difficile* ribotypes exhibited a complete reduction in log_10_ CFU on BHI agar plates and PBS following 20 min of exposure to a UV-C dose of 2208 mJ cm^−2^. The surface wipes with sporicidal properties demonstrated efficacy in reducing the number of *C. difficile* spores on the Formica, stainless steel, and plastic surfaces by 2.03–3.53 log_10_. The present study demonstrates that moist surfaces or liquids can enhance the efficacy of UV-C treatment in reducing *C. difficile* spores. This approach may be applicable to the surfaces of healthcare facilities and to water disinfection systems.

## 1. Introduction

*Clostridioides difficile* is an endospore-forming, anaerobic, Gram-positive bacterium. *C. difficile* infection (CDI) is a common cause of hospital-acquired diarrhoea. Its severity can range from mild to severe diarrhoea, pseudomembranous colitis, and toxic megacolon [1,2,3]. *C. difficile* is often characterised using PCR and there are several ribotypes (RTs) associated with CDI worldwide.

It is evident that spore formation by *C. difficile* plays a crucial role in survival in an aerobic atmosphere or resistance against extreme environmental stress (i.e., heat, desiccation, and exposure to disinfectants and antimicrobials), and disease transmission and persistence of CDI. The environment can harbour these highly infectious and resilient spores for up to five months [4,5]. The germination of *C. difficile* spores is initiated by specific environmental signals (germinants), which stimulate the reactivation of metabolic processes and vegetative cell growth [5]. The resilience of *C. difficile* spores has limited the effectiveness of current environmental decontamination strategies. Nevertheless, the effectiveness of ultraviolet-C (UV-C) light exposure on spores from toxigenic or nontoxigenic and antimicrobial-resistant *C. difficile* strains isolated from environmental sources has not been extensively studied so far, practically, on moist surfaces or in aqueous solutions. It is therefore imperative that advances are undertaken with the objective of reducing the environmental contamination of *C. difficile* spores in order to prevent patient infection.

UV light decontamination systems are becoming increasingly prevalent for the removal of *C. difficile* spores and other pathogens from hospital environments following terminal cleaning as well as in water distribution systems. The most germicidal wavelength of UV-C is in the region of 200–280 nm. Exposure to UV-C light results in DNA or RNA damage following photon absorption, causing the formation of thymine dimers between the bases thymine and cytosine [6,7,8]. Consequently, UV radiation causes microbial DNA to lose its physical and chemical structural properties, inhibits the production of metabolites for microbial growth, and prevents DNA replication, ultimately leading to microbial death [9]. UV-C light disinfection was originally carried out using conventional low-pressure mercury (Hg) discharge lamps, and the most efficient inactivation by UV-C could be achieved by increasing the number of photons at a wavelength of 254 nm [10,11]. The efficiency of emitted photons is significantly increased by the design and performance of high-pressure mercury lamps, which use electrodes capable of high voltage [10]. The wavelength of 254 nm is a crucial factor in the microbial disinfection mechanism of UV systems. It can be assumed that the wavelength of 253.7 nm is the most effective when using traditional mercury lamps for disinfection purposes [12]. The shorter wavelength of UV-C results in a higher light energy level than that of UV-B and UV-A. Consequently, UV-C exhibits a greater inactivation efficacy for a range of microorganisms in water phases [9,12,13]. In accordance with the mechanisms of inactivation, the disinfection of microorganisms in UV water treatment systems inhibits the replication and transcription of microbial genetic materials through the absorption of UV photons and the generation of polymers [12]. However, due to the distinct structural and biochemical characteristics of spores, including the presence of a protective chelate of dipicolinic acid (DPA) surrounding the DNA and an altered DNA configuration, spores demonstrate a remarkable resilience to radiation, with a resistance to between 10 and 50 times the radiation dose at 254 nm as compared to vegetative bacterial cells [14].

Sporicidal surface disinfection is strongly recommended for both outbreak and endemic settings [15]. To reduce the risk of *C. difficile* spore transmission, an effective treatment against spores on surfaces is necessary in healthcare facilities [16]. Therefore, the use of disinfectant wipes with sporicidal activity against *C. difficile* is recommended, which may reduce the incidence of healthcare-associated infections (HAIs) [17]. Commercially available wipes could be labelled as having “sporicidal” activity. The microbicides present in the wipes may have been tested for sporicidal activity using standard tests, such as the European standard EN 13704 [18], which is a sporicidal suspension test method developed for food hygiene, domestic, and institutional use. However, some manufacturers of surface disinfectants claim sporicidal activity of their products based on results obtained with *Bacillus subtilis* spores in suspension tests [19].

Both described methods offer the potential for the inactivation of *C. difficile* spores and reduced transmission through physical or chemical treatments. The present study therefore aimed to investigate the efficacy of UV-C 254 nm light in the inactivation of toxigenic or nontoxigenic and antimicrobial-resistant *C. difficile* spores of different PCR RTs (RT078, RT126, RT127, RT073, and RT001) at varying exposure times and UV-C doses on the surface of brain heart infusion (BHI) agar plates or in suspension [phosphate-buffered saline (PBS)]. It was hypothesised that the moist surface and aqueous suspensions would enhance the effectiveness of UV-C light in the deactivation of *C. difficile* spores. Furthermore, the efficacy of a sporicidal surface disinfectant against *C. difficile* spores on Formica, stainless steel, and plastic surfaces as representative materials in hospitals was evaluated.

## 2. Materials and Methods

### 2.1. C. difficile Strains

The tested environmental and reference *C. difficile* strains of five different PCR ribotypes used in the current study are listed in Table 1. Toxigenic and nontoxigenic *C. difficile* strains harbour different antimicrobial resistance genes encoding resistance to tetracyclines, aminoglycosides, streptothricins, beta-lactam antibiotics, or fluoroquinolones [20].

### 2.2. Preparation and Purification of C. difficile Spores

Spores of *C. difficile* strains were prepared and purified following the protocol described by Putnam et al. [22], with some modifications. Briefly, *C. difficile* strains were grown from glycerol stocks on BHI agar plates (Carl Roth GmbH & Co. KG, Karlsruhe, Germany). The cultures were then used to inoculate sporulation agar plates (70:30, a mixture of 70% sporulation medium and 30% BHI medium) [22], incubated anaerobically in anaerobic jars (Schuett-Biotec GmbH, Göttingen, Germany), and flushed with a mixture of gases (80% N_2_, 10% CO_2_, and 10% H_2_) at 37 °C for 5 days. The bacterial lawn was harvested in 1 mL or more of PBS (pH 7.4; NaCl 8 g/L, Na_2_HPO_4_ 1.44 g/L, KCl 0.2 g/L, and KH_2_PO_4_ 0.24 g/L) and centrifuged at 5000 rpm for 10 min. The supernatant was discarded, and the pellet was resuspended in PBS. This step was repeated twice in PBS and centrifuged as before. Finally, the pellet was resuspended in PBS and incubated overnight at 4 °C to lyse the vegetative cells. To eliminate any remaining vegetative cells, the spore suspension was subjected to heat shock at 70 °C for 20 min. The spores were then stored at 4 °C until use. The spore preparation was used to determine the number of viable spores per mL. Serial dilutions were carried out in PBS and the appropriate dilutions were plated on BHI agar plates supplemented with 0.1% (*w*/*v*) sodium taurocholate (ST) (Carl Roth GmbH & Co.KG, Karlsruhe, Germany). The inoculated plates were incubated anaerobically at 37 °C for 48 h. The resulting colonies were counted, and the colony-forming units (CFUs) per mL were determined. Additionally, the spore preparation was confirmed by determination of bright-phase spores using a microscope (Axioscope, Carl Zeiss Microscopy GmbH, Jena, Germany).

### 2.3. UV-C Radiation Source and Dose Measurement

A DESAG Lightbox UVIS (Desaga GmbH, Heidelberg, Germany) was used to conduct the UV-C treatment. This device consists of two 8 W low-pressure mercury lamps that emit UV-C light at a wavelength of 254 nm. The upper UV-C lamps were positioned 10 cm from the top of the sample. The sides of the Lightbox UVIS were made of plastic to minimise the diffusion of UV-C light.

**Figure 1:**
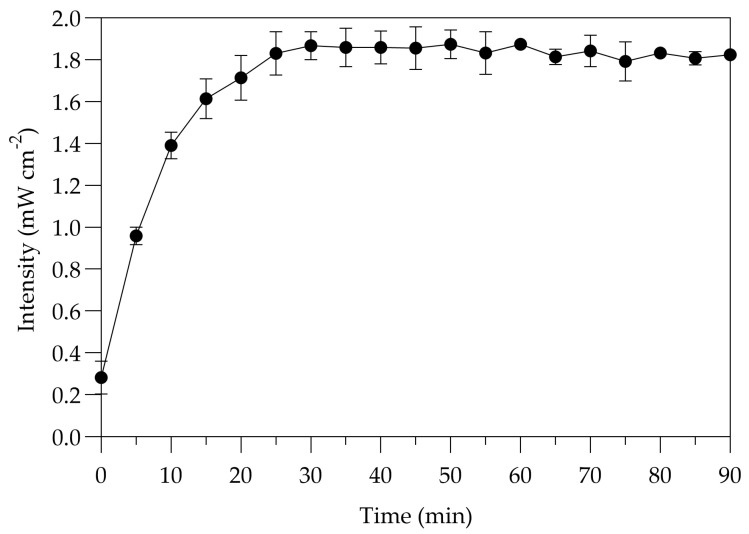
Intensities of UV-C 254 nm mercury lamps used in this study during the first 90 min exposure. Error bars represent the standard errors of the mean from three independent measurements.

The UV-C irradiance at 254 nm was measured using a digital optical power and energy meter (Model PM100D, THORLABS GmbH, Munich, Germany) during the first 90 min of exposure, before conducting the treatments. The radiometer was placed exactly in the centre of the tray where the inoculated sample was located, and three independent measurements of UV-C light were taken. The mean irradiance of the two mercury lamps was approximately 1.84 mW cm^−2^ after 30 min of exposure (Figure 1).

The UV-C devices were turned on for at least 30 min prior to all experiments. The UV-C dose for each cycle time was calculated using the mean of the irradiance measurements by using the following formula:

UV-C dose (mJ cm^−2^) = irradiance (mW cm^−2^) × exposure time (seconds) (1)

### 2.4. Inoculation, UV-C Treatments, and C. difficile Spore Recovery and Enumeration

The efficacy of UV-C 254 nm light against *C. difficile* spores from five different ribotypes on BHI agar plates or in PBS solution under controlled laboratory conditions was tested in two different sets of experiments.

**Figure 2:**
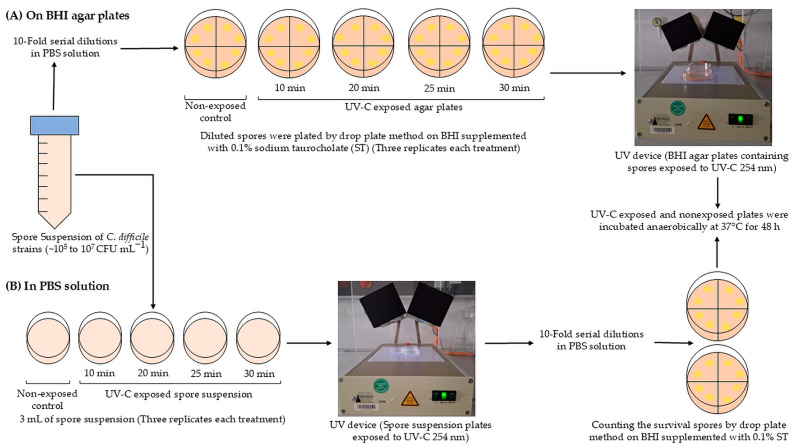
Schematic diagram of the experimental setup used to determine the efficacy of UVC 254 nm light on the spore germination ability of different *C. difficile* RT strains inoculated on the surface of BHI agar plates (**A**) and in PBS solution (**B**).

To expose BHI agar plates, 10-fold serial dilutions of *C. difficile* spore suspension in PBS were carried out with an initial concentration of *C. difficile* spores of approximately 10^5^–10^7^ CFU mL^−1^, dependent on the growth of respective RT strain. Afterwards, 20 µL (in duplicate) of appropriate dilutions (10^−1^ to 10^−5^) was placed on 100 mm diameter BHI agar plates supplemented with 0.1% ST using the drop plate method [23] (Figure 2A). The plates were air-dried in a safety cabinet at room temperature for 15 min. After the plates were dried, the inoculated BHI plates were placed under UV-C lamps at 254 nm and 10 cm for different exposure times (10, 20, 25, and 30 min). Nonexposed control samples were prepared using serial dilutions ranging from 10^−1^ to 10^−6^ CFU mL^−1^. Three independent experiments were conducted for each exposure time and nonexposed control samples. After irradiation, both the exposed and nonexposed plates were dried in a safety airflow cabinet for 15 min and incubated anaerobically in anaerobic jars at 37 °C for 48 h. After the incubation period, the number of colonies was counted to determine the CFUs per mL.

To expose the spore suspensions, 3 mL of spore suspension in PBS was transferred to empty Petri dishes (50 × 13 mm). The initial concentration of *C. difficile* spores was approximately 10^5^–10^7^ CFU mL^−1^, depending upon the growth of used RT strain. The inoculated Petri dishes were then placed 10 cm below the 254 nm UV-C mercury lamps for various exposure times as mentioned below (Figure 2B). The controls were not exposed to UV-C 254 nm light. The experiments were carried out in triplicate. Following irradiation, 10-fold serial dilutions were performed in PBS using the drop plate method. Then, 20 µL of the appropriate dilutions (10^−1^ to 10^−5^) were placed in duplicate on BHI agar plates supplemented with 0.1% ST. Serial dilutions between 10^−1^ and 10^−6^ CFU mL^−1^ were used for nonexposed control samples and plated as described above. The inoculated plates were dried in a safety airflow cabinet for 15 min and incubated anaerobically in anaerobic jars at 37 °C for 48 h. Following incubation, the number of colonies was counted, and the CFUs per mL was determined.

### 2.5. Sporicidal Surface Wipe Treatment

The efficacy of sporicidal peracetic acid-based surface wipes against spores of *C. difficile* strains (DS174, CF82, and RS151), as described above (Table 1) was determined. Mikrozid^®^ PAA wipes (Schuelke & Mayr GmbH, Norderstedt, Germany) were chosen for this study because they possess a spectrum of sporicidal and bactericidal activities. These wipes contain active ingredients, such as 0.07% peracetic acid, hydrogen peroxide, and acetic acid.

**Figure 3:**
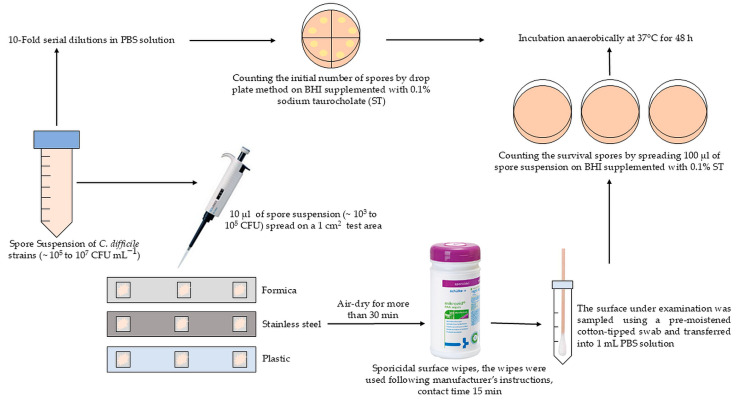
Schematic diagram of the experimental setup used to determine the efficacy of sporicidal surface wipes on the survival and germination ability of spores of different *C. difficile* RT strains.

The items (Formica, stainless steel, or plastic) were used as test surfaces for the disinfectant treatment test. Prior to utilization, all items were sterilized at 121 °C for 20 min. Ten microliters of spore suspension (containing approximately 10^3^ to 10^5^ CFU mL^−1^, depending on the growth of the RT strain) was inoculated onto each sample point and spread over a 1 cm^2^ test area (Figure 3). After inoculation, the test area was air-dried in the laminar airflow cabinet for more than 30 min. The wipes were used according to the manufacturer’s instructions for disinfecting medical devices and all types of surfaces. The inoculated test areas were thoroughly wiped with impregnated wipes to ensure complete wetting and allowed to take effect with a contact time of 15 min. After disinfection, a premoistened cotton swab was used to sample each test area. Each swab was placed in 1 mL of PBS and vortexed to release the spores. One hundred microliters of the suspension were plated onto BHI agar plates supplemented with 0.1% ST and incubated anaerobically at 37 °C for 48 h (Figure 3). After incubation, the number of colonies was counted, and the CFUs per cm^2^ was determined. The experiments were conducted with three replicates per surface for each strain.

### 2.6. Data Analysis

Each treatment (UV-C light or sporicidal surface wipe) was carried out in three replicates. The inactivation performance for *C. difficile* spores was determined by counting the CFUs, as described above, and presented as the mean log_10_ CFU reduction, which was calculated using the following equation:

Log_10_ CFU reduction = Log_10_ (B − A) CFU (2)

where B is the number of surviving and germinated spores before UV-C treatment (nonexposed control samples) and A is the number of surviving and germinated spores after UV-C irradiation (exposed UV-C samples).

The percentage reduction of spores on surfaces treated with a sporicidal surface wipe was calculated using the following formula:

Percentage reduction = B − A/B × 100%(3)

where B is the number of spores that survived and germinated before treatment, and A is the number of recovering and germinating spores on the test area after treatment.

Statistical analysis was performed using GraphPad Prism version 10, and a *p* value < 0.05 was considered significant, followed by Dunnett’s multiple comparison test to determine differences between exposed UV-C treatments and nonexposed controls. The Tukey test was used to determine differences between sporicidal wipe treatments on different surfaces.

## 3. Results

### 3.1. Efficacy of UV-C 254 nm Light in the Inactivation of C. difficile Spores at Various Exposure Times on the Surface of BHI Agar Plates and in PBS Solution

**Figure 4:**
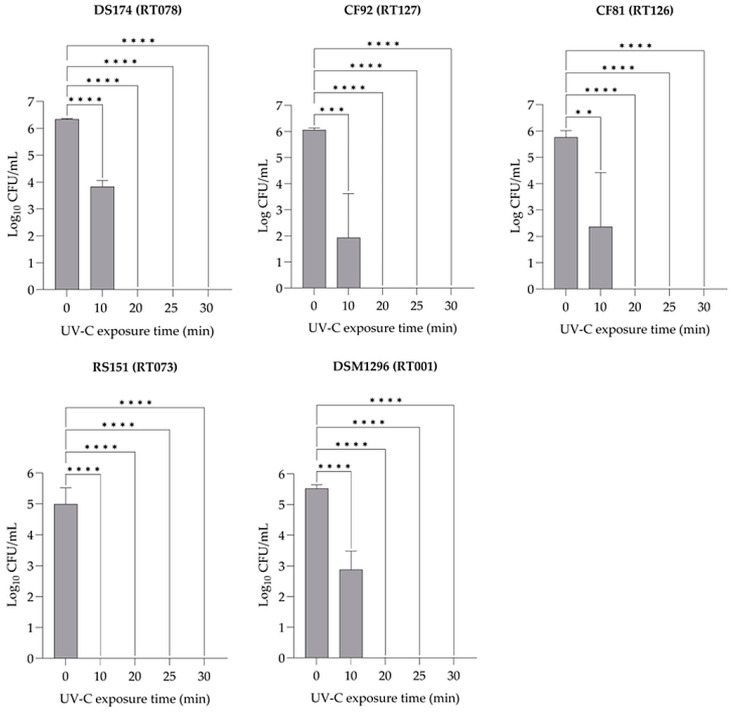
Recovery and germination ability of spores from five different *C. difficile* PCR ribotypes when exposed to UV-C 254 nm light on the surface of BHI agar plates after various exposure times. Each bar represents the mean of three replicates ± standard deviation. *p* < 0.0001 (****), *p* < 0.001 (***), *p* < 0.01 (**).

To evaluate the effectiveness of UV-C 254 nm light on the inactivation of *C. difficile* spores of different ribotypes, varying exposure times were applied. On average, 1.42 × 10^5^–2.17 × 10^6^ CFU mL^−1^ spores were inoculated on BHI agar plates and exposed to UV-C 254 nm light. The spore concentration of the *C. difficile* strains DS174 (RT078), CF81 (RT126), CF92 (RT127), RS151 (RT073), and DSM1296 (RT001) were recovered from nonexposed control samples on BHI plates, with counts of 6.34, 5.76, 6.06, 4.99, and 5.52 log_10_ CFU mL^−1^, respectively (Figure 4). After an exposure time of 10 min, the median number of CFUs decreased to 3.83, 2.37, 1.93, and 2.88 log_10_ for the toxigenic strains DS174, CF81, CF92, and DSM1296, respectively (*p* < 0.0001); the CF81 strain showed a significant decrease (*p* < 0.01). The recovery and germination of spores of the nontoxigenic strain RS151 completely decreased after 10 min of exposure (*p* < 0.0001). In general, the number of CFUs of all tested strains completely decreased after an exposure time of 20 min (*p* < 0.0001) (Figure 4).

**Figure 5:**
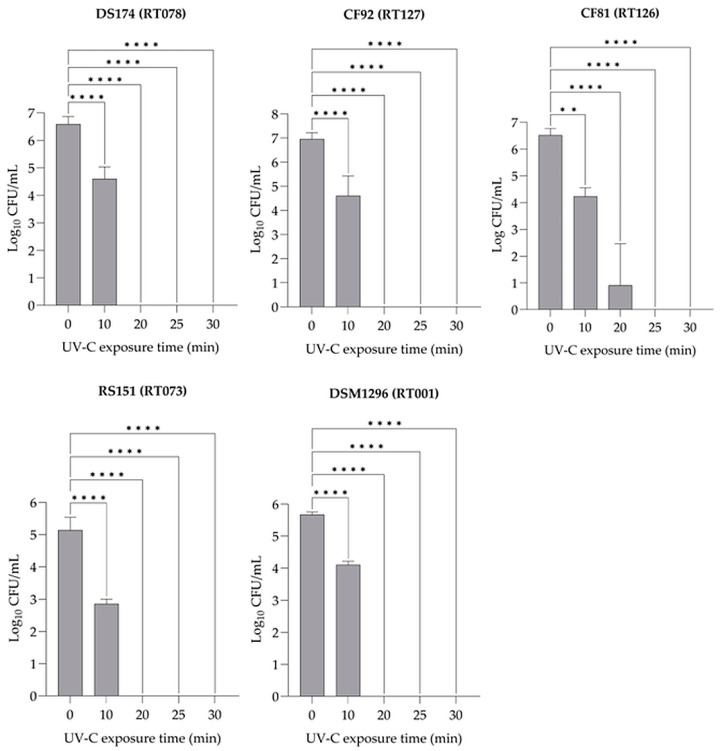
Recovery and germination ability of spores from five different *C. difficile* RT strains when exposed to UV-C 254 nm light in PBS solution for varying exposure times. Each bar represents the mean of three replicates ± standard deviation. *p* < 0.0001 (****), *p* < 0.01 (**).

To assess the efficacy of UV-C light at different exposure times on the inactivation of *C. difficile* spores in suspensions, an average of 1.75 × 10^5^–1.01 × 10^7^ CFU mL^−1^ spore suspension in PBS solution was placed into Petri dishes and exposed to UV-C 254 nm light. Spores of *C. difficile* strains DS174 (RT078), CF81 (RT126), CF92 (RT127), RS151 (RT073), and DSM1296 (RT001) recovered and germinated from the control samples in PBS solution, which were not exposed to UV-C light, showed the number of CFU mL^−1^ of 6.59, 6.52, 6.95, 5.14, and 5.67 log_10_, respectively (Figure 5). Following an exposure time of 10 min, the median number of CFUs decreased to 4.60, 4.23, 4.61, 2.86, and 4.10 log_10_ for the toxigenic and nontoxigenic strains, DS174, CF81, CF92, RS151, and DSM1296, respectively (*p* < 0.0001); the CF81 strain showed a significant decrease (2.28 log_10_; *p* < 0.01). After 20 min of exposure, the CFU count for the CF81 strain decreased to 0.90 log_10_ (*p* < 0.0001). The CFU count for all RT strains, except for strain CF81, decreased significantly after exposure times of 20 min and 25 min, respectively (*p* < 0.0001) (Figure 5).

### 3.2. Efficacy of the UV-C Dose for the Reduction of C. difficile Spores on the Surface of BHI Agar Plates and in PBS Solution

To evaluate the effectiveness of UV-C at a wavelength of 254 nm against spores of different *C. difficile* RT strains, different UV-C doses were applied. The spores were tested on the surface of BHI agar plates or in PBS solution with different spore densities at varying exposure times (10, 20, 25, and 30 min) and UV-C doses of 1014, 2208, 2760, and 3312 mJ cm^−2^. Nonexposed control samples were included.

**Figure 6:**
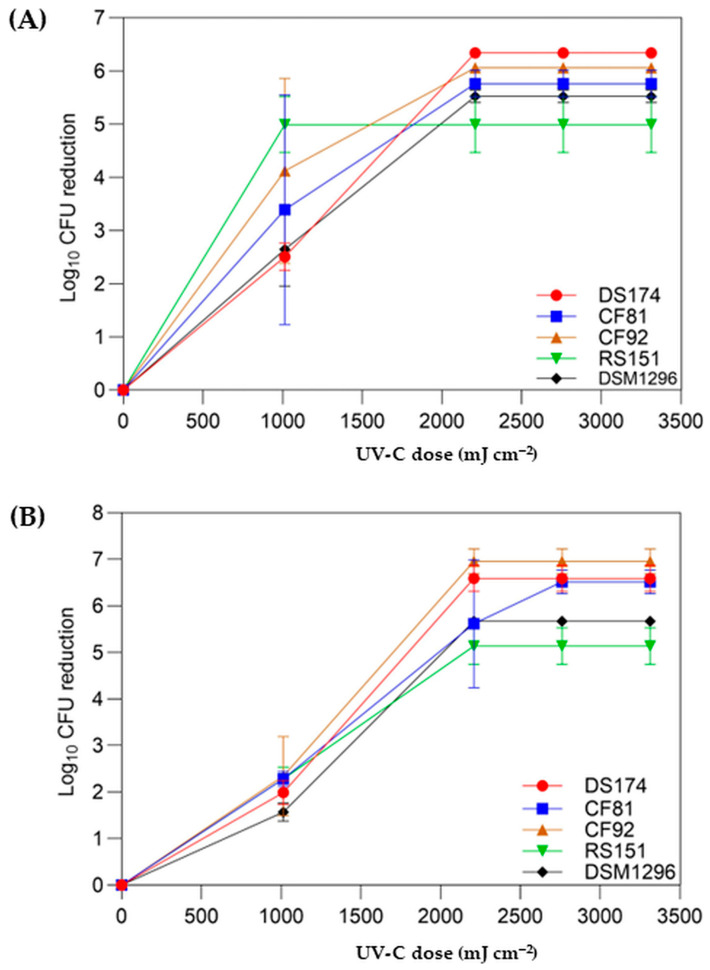
Log_10_ CFU reduction of spores from five different *C. difficile* ribotypes on the surface of BHI agar plates (**A**) and in PBS solution (**B**). Respective spores were exposed to UV-C light at various doses (1104 to 3312 mJ cm^−2^). The error bars represent the standard errors of the mean from independent experiments (*n* = 3).

After 10 min exposure of spores to UV-C 254 nm light on the surface of the BHI agar plates and subsequent germination and recovery, the log_10_ CFU reduction was calculated as 2.50, 3.39, 4.12, 4.99, and 2.64 for strains DS174, CF81, CF92, RS151, and DSM1296, respectively. All tested strains showed a complete log_10_ CFU reduction on the surface of BHI agar plates after being exposed to a UV-C dose of 2208 mJ cm^−2^ for 20 min, except for the nontoxigenic strain RS151 (RT073), which required a UV-C dose of 1014 mJ cm^−2^ for an exposure time of only 10 min to achieve complete reduction (Figure 6A, Table S1).

Exposure of spores to UV-C light in PBS solution for 10 min and their subsequent germination ability and growth led to a log_10_ reduction in CFUs of 1.99, 2.28, 2.34, 2.28, and 1.57 for strains DS174, CF81, CF92, RS151, and DSM1296, respectively. The log_10_ CFU reduction for strain CF81 was 5.62 after a 20 min exposure time (Figure 6B, Table S1). In general, the inactivation of *C. difficile* spores, resuspended in PBS solution requires only 20 min of exposure to an irradiance of ~1.84 mW cm^−2^ (corresponding to a dose of 2208 mJ cm^−2^) to achieve a complete reduction in the log_10_ CFU for all tested strains, except for strain CF81 (RT126), which required 25 min of exposure to a UV-C dose of 2760 mJ cm^−2^ (Figure 6B, Table S1). In summary, UV-C irradiation could effectively inactivate the number of *C. difficile* spores inoculated in PBS or on the surface of BHI agar plates.

### 3.3. Efficacy of Sporicidal Surface Wipes on the log_10_ CFU Reduction of C. difficile Spores on Different Surfaces

**Figure 7:**
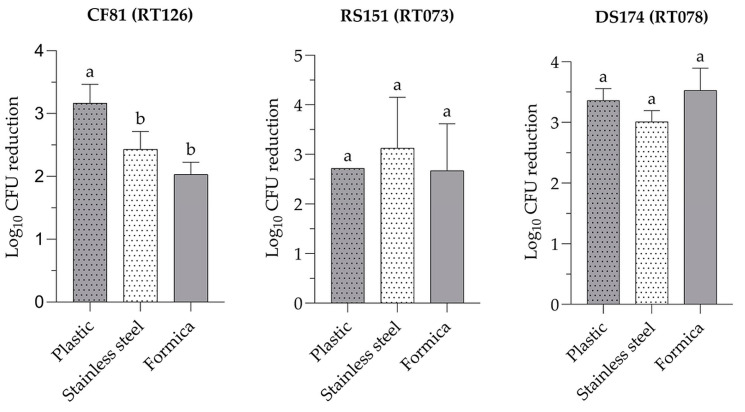
Inactivation of spores derived from different *C. difficile* ribotypes on different surfaces treated with sporicidal surface wipes. The mean of three replicates ± standard deviation is shown for each error bar. Significant differences (*p* < 0.05) between strains are indicated by different letters.

To evaluate the effectiveness of sporicidal surface wipes, spores of various *C. difficile* RT strains were inoculated onto a 1 cm^2^ test area on different surfaces at a concentration ranging from 10^3^–10^5^ CFU mL^−1^. The surfaces were then treated with sporicidal surface wipes according to the manufacturer’s instruction. The initial spore concentrations for strains DS174, RS151, and CF81 before treatment were 5.85, 3.72, and 5.27 log_10_ CFU mL^−1^, respectively. Treatment of plastic, stainless steel, and Formica surfaces with sporicidal surface wipes within 15 min of contact, resulted in an average log_10_ CFU reduction of [3.36 (95% CI 2.87–3.85), 3.01 (95% CI 2.56–3.47), and 3.53 (95% CI 2.62–4.44)], [2.72 (95% CI 2.72–2.72), 3.12 (95% CI 0.58–5.68), and 2.67 (95% CI 0.32–5.03)], and [3.17 (95% CI 2.42–3.91), 2.43 (95% CI 1.73–3.14), and 2.03 (95% CI 1.55–2.51)] for strains DS174 (RT078), RS15 (RT073), and CF81 (RT126), respectively (Figure 7, Table S2). Treatment of sporicidal surface wipes onto surfaces made of plastic, stainless steel, and Formica reduced the number of viable *C. difficile* spores by [57.52%, 51.54%, and 60.35%], [73.12%, 84.07%, and 71.81%], and [60.10%, 46.14%, and 38.56%] for strains DS174, RS151, and CF81, respectively. No significant differences in log_10_ CFU reduction of spores were observed between different surfaces inoculated with spores of strains RS151 and DS175, with the exception of surfaces inoculated with spores of the CF81 strain (*p* < 0.05) (Figure 7, Table S2).

## 4. Discussion

The efficacy of UV-C disinfection systems is dependent upon several factors, including the intensity of the radiation, the duration of the cycle, the position of the emitter, and the presence of physical barriers. The present study provides unique information on the efficacy of UV-C 254 nm light, UV-C dose, and exposure time on the inactivation of spores derived from toxigenic, nontoxigenic, and antimicrobial-resistant *C. difficile* strains of five different RTs on the surface of BHI agar plates or in PBS solution with different densities placed below UV-C mercury lamps at 10 cm. The spores of toxigenic *C. difficile* strains DS174 (RT078), CF81 (RT126), CF92 (RT127), and DSM1296 (RT001) were effectively inactivated from the surface of BHI agar plates after being exposed to a UV-C dose of 2208 mJ cm^−2^ for 20 min, whereas the nontoxigenic strain RS151 (RT073) required “only” a UV-C dose of 1014 mJ cm^−2^ and an exposure time of 10 min to achieve complete reduction. The results showed that also the inoculum concentration of *C. difficile* spores influences the treatment efficiency resulting in different susceptibilities to UV-C 254 nm light at varying UV-C doses and exposure times. In contrast, the average UV dose required for a 3 log_10_ CFU reduction in *C. difficile* spores was 16,000 mJ cm^−2^ for stainless steel and Formica laminates [24]. However, other studies using mobile devices have shown that doses ranging from 67,567 to >300,000 µW cm^−2^ resulted in a reduction of 3 log_10_ CFU [25,26]. Nerandzic et al. [27] reported that applying a Sterilray device at a radiant dose of 100 mJ cm^−2^ for five seconds reduced the recovery and germination of *C. difficile* spores by 4.4 log_10_ CFU on inoculated surfaces under laboratory conditions. In a further study, automated UV-C devices with irradiation for 40 min at four feet (~122 cm) from the devices reduced *C. difficile* spores germination by 3 log_10_ CFU cm^−2^ [28]. Boyce et al. [29] reported a 1.70–2.90 log_10_ CFU reduction in *C. difficile* spores germination when a population of 10^5^ CFU mL^−1^ was inoculated onto stainless steel carrier disks, which were placed in various areas within a hospital room, both in direct and indirect lines of UV-C and exposed to a light intensity of 22,000 μW cm^−2^ for 67.6 min. Rutala et al. [30] observed a 4 log_10_ CFU reduction in *C. difficile* spores germination on Formica laminate sheets when exposed to UV-C light with an intensity of 36,000 mW cm^−2^. After 50 min, a 99.8% reduction was observed.

A possible reason why previous studies required greater doses of UV-C compared to the current study is that the UV-C light source showed a higher distance to the test surfaces in those studies. Additionally, the spores were inoculated on the surface of agar plates in the present study, in contrast to previous studies, which may be enhanced the inactivation of spores. Moreover, these differences could be related to important factors involved in spore resistance to UV radiation, such as DNA saturation by α/β-type small acid-soluble spore proteins (SASPs), DNA repair during spore outgrowth, low core water content, and carotenoids in spore outer layers [8,31,32]. The SASPs are a family of proteins that are less than 100 amino acids in length and are highly conserved among all endospore-forming organisms. Most spore-forming bacteria encode two major α/β-type SASPs, namely SspA and SspB [31,33]. In this study, it is worth noting that the *C. difficile* strains used carried SASPs. The sequences of the strains have been submitted to NCBI GenBank under BioProject number PRJNA1011814 [20]. Nerber and Sorg [31] observed that *C. difficile* SspA is the primary contributor to the UV resistance of spores, while SspB plays a minor role in UV resistance. In addition, spores contain also numerous DNA repair proteins, including one that is specific to spores and which is active only with the presence of a UV photoproduct, known as SP [8].

However, it is difficult to compare the results of the abovementioned experiments described in different studies because the conditions under which they were conducted differed. This includes differences in the strains tested (i.e., toxigenic or nontoxigenic strains, RTs, source), spore load (counts of CFUs, 10^4^–10^6^, 10^5^–10^7^), UV-C exposure conditions (i.e., inoculum in liquid suspensions, placed on agar plates, or dried on solid surfaces), distance between the UV-C source and item, UV wavelength, exposure time, UV-C dose and temperature, and UV-C source. The effectiveness of UV-C treatments is influenced by several factors, including equipment specifications, UV-C processing parameters, product target characteristics, and microbial characteristics. Tande et al. [34] demonstrated that significant differences in disinfection occur due to large differences in UV light intensity across various surfaces.

Exposure to UV-C 254 nm light in PBS solution was used to inactivate spores of different *C. difficile* RT strains. Treating *C. difficile* spores in PBS solution with UV-C 254 nm light at 10 min exposure time (dose of 1104 mJ cm^−2^), resulted in CFU reduction of 1.57–2.34 log_10_. To achieve complete inactivation of the spores, a 20 min exposure time at a dose of 2208 mJ cm^−2^ was needed, except for strain CF81 (RT126), which required a 25 min exposure time at a dose of 2760 mJ cm^−2^. The variation in UV-C doses required to inactivate *C. difficile* spores may be due in part to clumping effects of the spores in the liquid suspension and the spore concentration. To the best of our knowledge, the efficacy of UV-C light on *C. difficile* spores in liquid suspensions has not been well investigated so far. Therefore, the required UV-C doses for reducing the log_10_ CFU of spores vary considerably due to differences in the laboratory techniques and conditions used by investigators. For instance, the methods for preparing *C. difficile* spores and their concentrations for testing differ substantially [24,30,35,36]. To achieve a specific log_10_ CFU reduction, a higher inoculum (e.g., 10^7^) may require greater UV-C doses than a lower inoculum (e.g., 10^5^) [37].

*C. difficile* contamination of clinical surfaces is common and has been linked to patient-to-patient transmission of the bacterium or its spores [4,38]. The “contaminated” hospital environment is a significant pathway for patients to develop CDI and it is known that *C. difficile* spores can survive for months [4,39]. Surface disinfectants can be used effectively against *C. difficile* spores on different surfaces, even under practical conditions, despite some manufacturer’s claim of sporicidal activity. The presented results provide more reliable data on the effectiveness of sporicidal disinfectants against *C. difficile* spores [40]. In the present study, the sporicidal surface wipes eliminated the spores of *C. difficile* strains on plastic, stainless steel, and Formica surfaces, with log_10_ CFU reductions of [3.01–3.53] for strain DS174 (RT078), [2.67–3.12] for strain RS15 (RT073), and [2.03–3.17] for strain CF81 (RT126). A previous study reported comparable outcomes using 10 different “sporicidal” wipes [41]. Nevertheless, the low spore load of *C. difficile* inoculated on the surfaces in the present study contrasts with the methodology described in previous studies. This approach was adopted due to the typically low levels of *C. difficile* that are quantified on the surfaces of clinical and other environments. A study conducted by Rutala et al. [42] demonstrated that sporicidal agents effectively eliminated more than 3.90 log_10_ of *C. difficile* spores, whereas wiping with non-sporicidal agents was found to remove more than 2.90 log_10_ of *C. difficile* spores. Their results also demonstrated that efficient transfer of *C. difficile* spores from contaminated to clean surfaces by sporicidal wipes can occur. Therefore, in clinical practice, it is important to prevent the spread of *C. difficile* spores on treated surfaces caused by wiping [43]. Additionally, the sporicidal wipe consistently achieved a log_10_ CFU reduction in *C. difficile* spores on rubber surfaces, ranging from 3.61 to 4.00, depending on the duration of contact [44]. Kenters et al. [45] reported that disinfecting cleaning wipes were more effective than spraying with the same active ingredients in reducing the CFUs of *C. difficile* RT010, RT014, and RT027 spores. The study also revealed that *C. difficile* spores of the toxigenic RT014 and RT027 strains are more challenging to eliminate than those of nontoxigenic RT010 strain. The log_10_ CFU reduction in *C. difficile* spores of the toxigenic DS174 (RT078) strain was greater than that against the toxigenic CF81 (RT126) and nontoxigenic RS151 (RT073) strains, depending on the surface type and inoculum concentration. Recently, sporicidal surface disinfectants have been demonstrated to be effective with at least a 4 log_10_ CFU reduction in the number of *C. difficile* spores in suspension and on surfaces at different contact times [46].

A limitation of the study was that all experiments were conducted under “clean” conditions, meaning no elevated concentrations of additional potential pathogens may be present on the surfaces in clinical settings. The presented experiments were performed under laboratory conditions (biosafety level 2) and obtained results of the efficiency were transferred to real conditions in healthcare facilities. This is particularly in regard to many variables, such as temperature, humidity, surface materials, UV-C dose, contact time, inoculum concentration of spores, airborne spores, organic load with the spores (e.g., faeces), and concentration of disinfectants. Controlling these variables in healthcare settings could be much more challenging than under laboratory conditions. A further limitation is the number of tested spores obtained from different strains, which are highly relevant in CDI outbreaks, including hypervirulent PCR ribotype strains (e.g., RT027, RT078) from humans. Nevertheless, the results obtained under precisely defined conditions provide valuable information on the efficiency of the disinfection measures presented in the clinical and veterinary environments.

## 5. Conclusions

The present study confirms the sporicidal efficiency of UV-C 254 nm light and suggests potential mechanisms for enhancing its activity on moist surfaces or in aqueous suspensions. The findings of the current study demonstrate that UV-C mercury lamps are effective in the inactivation of *C. difficile* spores, thereby establishing it as a potential solution for the enhancement sanitation processes in a range of environments. First conclusions from five *C. difficile* strains differing in ribotypes and antimicrobial susceptibility shows that further research is required to evaluate the efficacy of UV-C 254 nm light on a larger scale, particularly in the context of hypervirulent ribotypes of *C. difficile* spores. The utilize of sporicidal surface wipes has been demonstrated to be an effective methodology for the reduction of the number of spores of *C. difficile* strains on a range of surfaces. Consequently, the regular use of sporicidal wipes on “critical” surfaces in hospitals and nursing homes can significantly reduce the risk of *C. difficile* transmission from contaminated surfaces. Although the use of sporicidal wipes could provide additional control of the microbial burden on surfaces, it is necessary to test the efficacy and label of antimicrobial and sporicidal wipes before they are released into the market. This is because unsuitable wipes may be used in healthcare applications without showing the expected effect.

## Figures and Tables

**Table 1:** Environmental and reference *C. difficile* strains used in the present study.

Strain ID	PCR-Ribotypes	Toxin Genes	Source	Reference
DS174	RT078	TcdA, TcdB, CDT	DSS-S	[20,21]
CF81	RT126	TcdA, TcdB, CDT	CF	
CF92	RT127	TcdA, TcdB, CDT	CF	
RS151	RT073	-	RS	
DSM1296	RT001	TcdA, TcdB	Human	DSM

DSS-S: digested-sewage sludge amended soil, CF: calf faeces, RS: raw sewage. DSM: Deutsche Sammlung von Mikroorganismen (Leibniz Institute, German Collection of Microorganisms, Braunschweig, Germany).

## Data Availability

Data are presented in the article and Supplementary Materials.

## Supplementary Materials

The following supporting information can be downloaded at: https://www.mdpi.com/article/10.3390/pathogens13110965/s1, Table S1: Effectiveness of varying UV-C dose (mJ cm^−2^) on the log_10_ CFU reduction of antimicrobial-resistant *C. difficile* spores on BHI agar plates and in PBS solution; Table S2: Efficiency of sporicidal surface wipes on the log_10_ CFU reduction of *C. difficile* spores on different surfaces. Significant differences (*p* < 0.05) between strains are indicated by different letters.
