# Supplementary material for: Efficacy of UV-C 254 nm Light and a Sporicidal Surface Disinfectant in Inactivating Spores from Clostridioides difficile Ribotypes In Vitro
Source: Pathogens. 2024 Nov 5;13(11):965. doi: 10.3390/pathogens13110965 (PMC11597166; doi:10.3390/pathogens13110965)
Supplement: Supplementary file 1 [file pathogens-13-00965-s001.zip › Supplementary Materials.pdf]

Supplementary information

Efficacy of UV-C 254 nm Light and a Sporicidal Surface Disinfectant in the Inactivation of Spores from *Clostridioides difficile* Ribotypes In Vitro

Khald Blau and Claudia Gallert

Table S1: Effectiveness of varying UV-C dose (mJ cm^{-2}) on the log_{10} CFU reduction of antimicrobial-resistant *C. difficile* spores on BHI agar plates and in PBS solution.

Treatments	Exposure time (S)	UV-C dose (mJ cm^{-2})	DS174 (RT078)		CF81 (RT126)		CF92 (RT127)		RS151 (RT073)		DS1296 (RT001)	
			Mean log_{10} CFU reduction	95% CI	Mean log_{10} CFU reduction	95% CI	Mean log_{10} CFU reduction	95% CI	Mean log_{10} CFU reduction	95% CI	Mean log_{10} CFU reduction	95% CI
On BHI agar plates	10	1,014	2.50	1.80-3.15	3.39	-1.97-8.76	4.12	-0.21-8.45	4.99	3.69-6.30	2.64	0.92-4.36
	20	2,208	6.34	6.26-6.41	5.76	5.14-6.38	6.06	5.86-6.25	4.99	3.69-6.30	5.52	5.23-5.81
	25	2,760	6.34	6.26-6.41	5.76	5.14-6.38	6.06	5.86-6.25	4.99	3.69-6.30	5.52	5.23-5.81
	30	3,312	6.34	6.26-6.41	5.76	5.14-6.38	6.06	5.86-6.25	4.99	3.69-6.30	5.52	5.23-5.81
In PBS solution	10	1,014	1.99	1.34-2.64	2.28	1.88-2.69	2.34	0.23-4.46	2.28	1.66-2.90	1.57	1.07-2.06
	20	2,208	6.59	5.89-7.28	5.62	2.20-9.04	6.95	6.28-7.62	5.14	4.15-6.13	5.67	5.46-5.89
	25	2,760	6.59	5.89-7.28	6.52	5.89-7.15	6.95	6.28-7.62	5.14	4.15-6.13	5.67	5.46-5.89
	30	3,312	6.59	5.89-7.28	6.52	5.89-7.15	6.95	6.28-7.62	5.14	4.15-6.13	5.67	5.46-5.89

CI: confidence interval.

Table S2: Efficiency of sporicidal surface wipes on the log_{10} CFU reduction of *C. difficile* spores on different surfaces. Significant differences (p<0.05) between strains are indicated by different letters.

Treatments	DS174 (RT078)		CF81 (RT126)		RS151 (RT073)	
	Mean log_{10} CFU reduction	95% CI	Mean log_{10} CFU reduction	95% CI	Mean log_{10} CFU reduction	95% CI
Plastic	3.36a	2.87–3.85	3.17a	2.42–3.91	2.72a	2.72–2.72
Stainless steel	3.01a	2.56–3.47	2.43b	1.73–3.14	3.13a	0.58–5.68
Formica	3.53a	2.62–4.44	2.03b	1.55–2.51	2.67a	0.32–5.03

CI: confidence interval.
